# NeuroMark dFNC: Transferable Dynamic-State Templates for Subject-Specific and Frequency-Resolved Chronnectomics

**DOI:** 10.64898/2026.10.12.758630

**Published:** 2026-10-17

**Authors:** M. Moein Esfahani, Masoud Seraji, Sepehr Salem, Kyle M. Jensen, Zening Fu, Tulay Adali, Yu-Ping Wang, Vince Calhoun

## Abstract

Dynamic functional network connectivity (dFNC) commonly identifies recurring connectivity states from resting-state fMRI, but states derived independently in each dataset lack guaranteed correspondence across studies and cannot be directly estimated in new participants. We introduce NeuroMark dFNC, which extends the NeuroMark principle of prior-guided network estimation to dynamic connectivity by using reproducible reference states as priors for estimating corresponding subject-specific states. Reference-state templates were derived from UK Biobank using windowed functional network connectivity and time-frequency moments (TFMs), with split-sample reproducibility evaluated for k-means, independent component analysis (ICA), and principal component analysis (PCA) template-generation approaches. Constrained ICA then used the templates to estimate subject-specific state patterns and continuous loadings in independent FBIRN schizophrenia and ADNI Alzheimer's disease cohorts. ICA loadings were calibrated by their contribution to reconstruction of the original feature matrix. Fractional occupancy and conditional mean dwell time were calculated from the state with the largest absolute calibrated loading at each observation. The predefined templates avoided the need for dataset-specific state discovery and post-hoc state matching. In both clinical cohorts, the analysis identified state-dependent, distributed edgewise differences and differences in selected temporal summaries. Nominal frequency-resolved findings varied across states and moment-frequency bins and were treated as exploratory. NeuroMark dFNC provides a common state space for cross-cohort comparison while retaining subject-specific connectivity patterns and continuous state expression.

